# A Gradient-Projected Model for Image Denoising

**DOI:** 10.3390/s26010013

**Published:** 2025-12-19

**Authors:** Yuming Wen, Yu Liu, Zhaozhi Liang, Guangjun Xu, Cong Lin, Guancheng Wang

**Affiliations:** College of Electronic and Information Engineering, Guangdong Ocean University, Zhanjiang 524088, China

**Keywords:** image denoising, gradient-projected function (GPF), optimization, deep learning

## Abstract

Digital images are prone to various forms of noise during acquisition, which can distort structural information and hinder subsequent processing. This work proposes AuroraNet, a denoising framework that extends the dual-branch design of DudeNet and integrates a Gradient-projected Function (GPF) optimizer to enhance training stability and preserve fine-scale image features. We evaluated the model on two real-world noisy image datasets to examine its robustness under different noise conditions. AuroraNet achieved an average PSNR of 35.59 dB on the first dataset and 38.40 dB on the second, together with an SSIM of 0.9633 in the latter. Across both benchmarks, AuroraNet consistently delivered higher reconstruction quality than several established models and the baseline DudeNet. Although R-REDNet produced the highest overall scores on one of the datasets, AuroraNet attained comparable performance while using a much smaller amount of parameters, underscoring its efficiency and practical value. These results indicate that AuroraNet offers a balanced solution for real-world image denoising, providing strong denoising capability without sacrificing computational economy.

## 1. Introduction

Image denoising plays an indispensable role in many fields such as image restoration, medical imaging, computer vision, digital photography, and various image-related applications. However, images are easily polluted by noise during the acquisition process, especially in complex environments such as low illumination, cloud cover, sensor failure, atmospheric disturbance, etc. Generally speaking, the polluted noises including additive white Gaussian noise (AWGN) [1], impulse noise [2], quantization noise [3], Poisson noise [4], and speckle noise [5]. Among these noises, AWGN mainly acts on analog circuits, while other types of noises are mainly due to manufacturing defects, incorrect coding and insufficient number of photons in the image acquisition process [6]. The above-mentioned types of noise not only reduce the visual quality of the image but also seriously affect the subsequent analysis and interpretation of the image, resulting in a decrease in feature classification accuracy, a decrease in target recognition accuracy, and even key decision-making errors. Therefore, image denoising has been a top priority in the field of image processing.

Traditional image denoising methods, such as filtering algorithms and statistical models [7], usually have difficulty removing noise effectively while retaining the detailed information in the image completely. These methods are prone to causing blurring of image edges and loss of texture information, which ultimately leads to the loss of valuable information. In addition to this, denoising methods based on deep learning have gradually become mainstream [8], but deep neural networks face problems such as gradient vanishing and local optimality during training, which lead to a poor model denoising effect, insufficient generalization ability, or the need to consume a large amount of computational resources. Therefore, how to design an image denoising model that is both efficient and accurate under complex noise environments remains a technical challenge. To address the aforementioned challenges in existing technologies, this paper proposes a novel deep learning framework for image denoising, named AuroraNet. AuroraNet is built upon the dual denoising network (DudeNet) architecture [9] and employs an innovative Gradient-projected Function (GPF) optimizer. This integration is designed to leverage the strengths of the GPF optimizer in mitigating issues such as gradient vanishing while simultaneously utilizing DudeNet’s capability in extracting fine image details, thereby enhancing the overall denoising performance.

The highlights of this work are:

1. A novel image denoising model is proposed, which combines the DudeNet architecture with an optimizer based on GPF to effectively handle complex noise patterns.
2. The GPF optimizer enhances training stability by mitigating vanishing and exploding gradients, enabling better convergence and improved denoising performance.
3. Extensive experiments show that AuroraNet outperforms models using the Adam optimizer and other state-of-the-art methods, achieving higher PSNR and SSIM while preserving fine image details.

## 2. Related Work

### 2.1. Traditional Denoising Methods for Images

Prior to the emergence of deep learning techniques, the denoising of images primarily depended on traditional signal processing and statistical modeling approaches. While these methods demonstrate certain capabilities in noise suppression, they frequently encounter difficulties in effectively preserving image details during the denoising process. Among traditional denoising techniques, spatial domain filtering remains one of the most widely used approaches. Mean filtering operates by computing the average gray value within a pixel’s neighborhood to smooth the image. Radhika et al. recently introduced an adaptive optimally weighted mean filter that dynamically adjusts neighborhood weights based on local gradient features [10]. While easy to implement, this method often results in image blurring and the loss of high-frequency details. In contrast, median filtering replaces the central pixel with the median of its neighbors, offering better suppression of salt-and-pepper noise. Guo et al. developed an optimized weighted median filter with adaptive thresholding, achieving an improvement in COVID-19 CT image processing [11]. However, median filtering typically incurs higher computational cost and may still introduce some blurring. To better preserve image details, adaptive filters such as the Wiener filter have been developed. Liu et al. proposed a windowed variation kernel Wiener filter that integrates spatial-variant kernels with frequency-domain constraints which improves edge retention via spatial-frequency adaptive mechanisms [12]. Despite their effectiveness, such adaptive filters are often complex in both design and implementation.

In addition to spatial domain techniques, transform domain filtering represents a major class of traditional image denoising methods. These approaches first project the image into a transformed domain—such as the frequency domain—where noise suppression is performed before converting the result back into the spatial domain. The Fourier transform is commonly used to analyze the frequency characteristics of images. Paska M P explored improved image denoising through fractional anisotropic diffusion and resolution-tailored differentiation in the Fourier domain [13]. However, due to the global nature of the Fourier basis, these methods often fall short in effectively handling non-stationary or irregular noise patterns. By contrast, the wavelet transform, with its inherent multi-resolution analysis capability, offers improved performance for non-smooth noise [14]. Despite these advantages, wavelet-based methods tend to be computationally intensive and may introduce artifacts, particularly edge blurring, during the reconstruction process [15].

Furthermore, statistical modeling methods have found widespread application in image denoising. The total variation (TV) regularization approach suppresses noise by constraining the image’s total variation. Extending this concept, Bi et al. proposed a primal-dual hybrid gradient algorithm that incorporates both overlapping group sparsity and fractional-order total variation. This advanced regularization scheme aims to leverage the benefits of TV in edge preservation while the group sparsity promotes structural regularities, potentially offering finer control over smoothing and detail retention compared to standard TV methods [16]. Additionally, the Markov random field (MRF) model assumes specific dependencies among image pixels and employs this model to estimate the original image. Cao et al. proposed an MRF model that implements multi-scale Gibbs sampling that adaptively controls smoothing intensity [17]. However, the model assumptions may not always align with practical scenarios, and the method entails high computational complexity. Similar to other approaches, MRF-based methods tend to over-smooth images, resulting in blurred edges [18]. These inherent limitations make it challenging for traditional methods to simultaneously achieve effective noise removal and detail preservation while maintaining good adaptability in complex scenarios.

Although traditional image denoising methods can suppress noise to some extent, they face a fundamental challenge in balancing denoising effectiveness with detail preservation. These approaches often yield unsatisfactory results when confronted with complex noise patterns. To overcome these limitations, many researchers have recently shifted toward deep learning-based denoising methods, which have demonstrated significant advances.

### 2.2. Deep Learning-Based Approaches for Image Denoising

Traditional numerical techniques for image denoising, while effective under specific assumptions, often struggle to handle complex and diverse noise patterns encountered in real-world scenarios. In contrast, neural network models have emerged as powerful alternatives, capable of learning flexible and highly non-linear mappings from noisy to clean images. Their strong generalization and adaptability have enabled remarkable performance not only in standard image restoration tasks but also in downstream applications such as robot perception, autonomous navigation, and other control-related auxiliary tasks where accurate visual information is critical [19,20,21,22]. An early and influential work in this field is the DnCNN (denoising convolutional neural network) proposed by Zhang et al., which integrates deep residual learning with batch normalization [23]. DnCNN demonstrates strong performance in removing additive white Gaussian noise, establishing a solid foundation for subsequent CNN-based denoising models. In addition, encoder–decoder architectures, especially those inspired by U-Net’s symmetrical design and skip connections, have become highly effective in image denoising. For example, Ni et al. proposed an image denoising method based on a U-Net neural network specifically for high-voltage insulator damage images [24], which has become popular. U-Net utilizes a symmetrical architecture with contracting and expansive pathways bridged by skip connections. This design efficiently captures multi-scale contextual features and reconstructs fine-grained details, making it well suited for diverse image restoration tasks—including denoising across multiple imaging modalities. In addition, other deep learning approaches for image denoising have also been extensively explored. For example, generative Adversarial Networks (GANs) proposed by Goodfellow et al. have been used for image denoising tasks [25]. Boucherit et al. designed an enhanced residual encoder–decoder network (R-REDNet) that replaces additive skip connections with averaging operations and adopts an iterative refinement strategy, which achieved superior PSNR and SSIM metrics on real-world noisy image datasets [26]. In recent years, transformer-based models have also attracted much attention, especially in image denoising [27,28,29,30]. For example, a heterogeneous window-based Transformer is introduced by Tian et al., which effectively balances long–short distance modeling and computational efficiency for image denoising [29]. By integrating global contextual interactions with lightweight local refinement, the proposed approach enhances structural recovery while significantly reducing inference time. In addition, Zhou et al. designed LIDFormer, a lightweight image denoising Transformer that achieves an improved balance between restoration quality and computational efficiency by combining wavelet-based dimensionality reduction, complementary feature reuse, and a triple-head attention mechanism [30].

Current deep learning-based denoising methodologies face three persistent technical challenges. First, the training process remains vulnerable to gradient instability issues, including both vanishing and exploding gradients. Second, existing models continue to underperform in preserving critical image details during denoising operations. Third, an inherent tendency toward excessive smoothing frequently compromises output quality.

### 2.3. Evolution of Optimization Algorithms in Deep Learning Models

Optimization of deep neural networks is key to their success, but it is challenging due to their complex, high-dimensional, and non-convex loss landscapes [8]. Therefore, effective optimized algorithms are crucial for training high-performance models.

The underlying method is stochastic gradient descent (SGD), which updates parameters using gradients computed over mini-batches of data [31]. While computationally efficient, vanilla SGD converges slowly and can have difficulty handling saddle points or sharp minima in the loss graph [32]. To improve SGD, momentum was introduced, which accelerates convergence by accumulating a velocity vector in the direction of the continuous gradient [33,34]. Moreover, the Nesterov accelerated gradient (NAG) method further improves momentum by computing the gradient after a “lookahead” momentum step [35]. Adaptive learning rate methods have made significant progress, which adjusts the learning rate of each parameter based on the squared gradient history, but its monotonically decreasing learning rate can stop learning prematurely [36]. Furthermore, Liu et al. employed a non-linear activation function on the gradients to improve training stability [37]. While first-order methods such as Adam dominate due to their computational efficiency, second-order methods that exploit Hessian information can theoretically provide faster convergence by exploiting curvature information. However, for large-scale models commonly found in deep learning, computing and storing the Hessian matrix is often computationally expensive [38].

## 3. Method

### 3.1. Overall Network Structure

**Figure 1:**
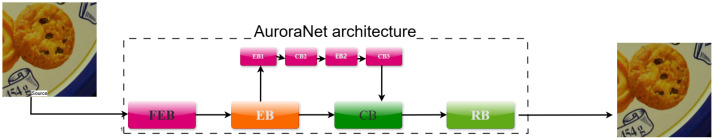
Network architecture of the AuroraNet.

AuroraNet is a novel image denoising model, whose core architecture is based on the DudeNet framework [39]. This network employs an end-to-end deep convolutional neural network design, aimed at systematically processing input images through a series of meticulously crafted modules to achieve efficient denoising. The entire network primarily consists of four key components that work collaboratively to complete the entire denoising process, from feature extraction to image reconstruction: Feature Extraction Block (FEB), Enhancement Block (EB), Compression Block (CB), and Reconstruction Block (RB). We denote the network’s input as the noisy image *Y*, and the final denoised output as *X*. The overall architecture of AuroraNet is visually represented in Figure 1, which illustrates the data flow and the key components of the network.

The data flow within the network is as follows: First, the Feature Extraction Block (FEB) F(·) receives the input noisy image *Y* and extracts multi-scale, multi-level initial feature representations, denoted as $F_{\mathrm{feat}}$. Next, these features $F_{\mathrm{feat}}$ are fed into the Enhancement Block (EB) E(·), which is responsible for deep fusion and reinforcement of the features to capture fine image details and suppress noise, generating enhanced features $E_{\mathrm{feat}}$. Subsequently, the Compression Block (CB) C(·) performs dimensionality reduction and refinement on the enhanced features $E_{\mathrm{feat}}$, aiming to preserve the most critical discriminative information while reducing redundancy, outputting compressed features $C_{\mathrm{feat}}$. Finally, the Reconstruction Block (RB) R(·) utilizes these processed features $C_{\mathrm{feat}}$ along with the original input *Y* for residual learning to reconstruct a high-quality denoised image X. The overall network process can be conceptualized as

(1) $$X=R\left(Y,C_{\mathrm{feat}}\right)=Y-C_{\mathrm{feat}}.$$

The detailed internal architecture and specific operations of each module will be discussed in depth in subsequent sections.

### 3.2. Feature Extraction Block

The FEB, as the first layer of DudeNet, is the foundation for effective denoising and high-quality image reconstruction. FEB aims to extract highly discriminative multi-scale and multi-resolution feature representations from the input noisy image *Y*, which provides the key feature space for subsequent operations such as non-linear mapping, noise suppression, and detail recovery. To achieve this goal, FEB adopts a well-designed two-branch parallel structure with two complementary sub-networks, FEBnet1 and FEBnet2, in order to organically combine global context-awareness and local detailed texture capture: FEBnet1, as the first layer of the module; the network can be roughly divided into three sub-models; Conv + BN + ReLU, Dilated Conv + BN + ReLU, and Conv.; and the specific one that performs the dilation convolution is Conv + BN + ReLU. all units will be carried out in the proposed sparsification mechanism, while all sub-models have their own roles and are effective in different layers. The second to the sixteenth layers constitute the sparsification mechanism of FEBnet1. Therefore its formula is roughly

(2) $$FEB_{1}=C\left(CBR_{3}\left(S\left(CBR_{1}\left(Y\right)\right)\right)\right),$$

where $CBR_{1}\left(\cdot \right)$, S(·), $CBR_{3}\left(\cdot \right)$, and C(·) represent the functions of Conv + BN + ReLU, designed sparse mechanism, three Conv + BN + ReLU, and one 3 × 3 convolution, respectively. This is converted to $F_{1}\left(Y\right)$ = $FEB_{1}$ via (1). Since it focuses on extracting the global structural information and long-range dependencies of the image and introduces techniques such as sparse connected null convolution (Atrous Convolution), it effectively expands the perceptual domain while reducing the computational complexity, thus improving the network’s ability to perceive the overall structure of the image.

The second sub-module FEBnet2 is mainly composed of Conv + ReLU and CB1. Its formulation can be mainly represented as

(3) $$FEB_{2}=C_{1}\left(CR_{15}\left(Y\right)\right),$$

where $CR_{15}$ represents the stacking of fifteen Conv + ReLU functions. This module is designed to capture local patterns and intricate features from the image. The number of layers, 15, was chosen after ablation studies, as it provides an optimal balance between capturing essential details for denoising and maintaining computational efficiency. Adding more layers did not result in significant performance gains but increased the computational burden. Therefore, we selected 15 layers to achieve the best performance-to-efficiency ratio.

Finally, the FEB module splices and fuses the feature maps extracted by FEBnet1 and FEBnet2 in the channel dimension to form a multi-scale feature representation that takes into account the global context and local details, which provides a more comprehensive and reliable feature base for the subsequent modules and improves the overall denoising performance.

### 3.3. Enhancement Block

The Enhancement Block EB is located in the middle part of DudeNet, which is also composed of two parts (EB1 and EB2), through which the learning function of the design network is enhanced to cope with various types of noise. Its overall function is to adaptively adjust and non-linearly map the multi-scale features extracted by the FEB, effectively integrating the original image information, thus improving the robustness of the model to complex noise and enhancing the reconstruction of image details.

Specifically, EB1 contains three parts: fusion part, batch normalization (BN), and ReLU. The fusion part is responsible for adaptively adjusting the channel size according to the features output from the FEB, stabilizing the training process by BN to reduce undesirable effects, and finally introducing linear features into the non-linearities by means of the ReLU activation function. EB2, on the other hand, fuses the original noise-bearing image with the features processed by CB2 by means of residual learning, so as to supplement the detail information of the original image at the feature level, suppress possible artefacts, and retain more high-frequency components. Through the synergy of the two sub-modules, the EB module achieves effective enhancement of features and fine recovery of image details, providing better quality input features for the subsequent compression and reconstruction modules.

### 3.4. Compression Block and Reconstruction Block

In the DudeNet architecture, the CB and the RB reflect in-depth consideration of feature representation redundancy and information reconstruction strategies. Considering that deep neural networks usually generate a large amount of redundant information in the feature extraction stage, applying it directly to image reconstruction will bring a huge computational burden and potential risk of overfitting, it will be divided into three parts, CB1, CB2, and CB3. They are located in the FEB as well as in the different layers of the EB, respectively. The design objective is to achieve effective purification and filtering of feature representations by compressing the feature dimensions layer by layer, so as to retain the discriminative information that is most critical for image denoising.

### 3.5. Gradient-Projected Function

GPF is an algorithm used in deep learning to adjust the magnitude of gradients. It dynamically amplifies small gradients and limits large gradients, ensuring the stability of gradient updates and addressing gradient-related issues during neural network training, thereby improving the performance of deep neural networks.

Assume that there are *n* samples $\left\{x^{\left(1\right)},\ldots ,x^{\left(n\right)}\right\}$ in a training batch, and the corresponding labels are $\left\{y^{\left(1\right)},\ldots ,y^{\left(n\right)}\right\}$. $M(x^{\left(i\right)};w^{\left(i\right)})$ is the output of the training model for each input sample $x^{\left(i\right)}$ during each iteration, where $w^{\left(i\right)}$ is the weight vector. η represents the learning rate, and $\mu_{m}$ is the momentum coefficient in the optimizer. L(·) represents the loss function. Next, calculate the gradient *g*, with the formula denoted as

(4) $$\overset{ˇ}{g_{n}}=\nabla_{\omega }\sum_{i=1}^{n}\frac{L\left(M\left(X^{\left(i\right)};\omega^{\left(i\right)}\right),y^{\left(i\right)}\right)}{n},$$

**Figure 2:**
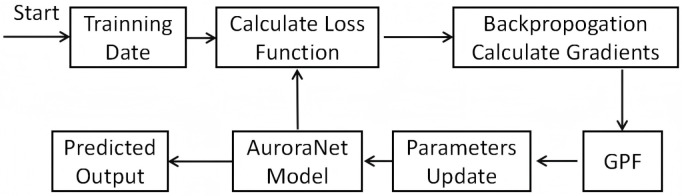
AuroraNet training process with GPF.

The complete training procedure (as shown in Figure 2) with GPF can be formalized as an iterative gradient optimization process. $g^{\prime }\left(g_{n}\right)$ indicates a gradient adjustment function, defined as a mapping from *n*-dimensional real space to *n*-dimensional real space, which transforms the gradient vector. This adjustment function $g^{\prime }\left(g_{n}\right)$ can take different functional forms. $w_{k}$ is the weight vector at the *k*-th iteration, initialized to 0.

Repeat the following procedure until the loss value computed by the loss function becomes sufficiently small and stable. After the iteration, using the gradient was that activated by the GPF algorithm replaces the original gradient.

$$\left\{\begin{array}{cc} \overset{ˇ}{g_{n}} & =\nabla_{\omega }\sum_{i=1}^{n}\frac{L\left(M\left(X^{\left(i\right)};\omega^{\left(i\right)}\right),y^{\left(i\right)}\right)}{n}\\ g_{n} & =\mu_{m}g_{n-1}-\eta {\hat{g}}_{n}\\ g_{n}^{\prime } & =g^{\prime }\left(g_{n}\right)\\ \omega_{n} & =\omega_{n-1}+g_{n}^{\prime } \end{array}\right.$$

For different types of GPF, the formula for $g^{\prime }\left(g_{n}\right)$ varies as follows:

- Arctan-type GPF:
  (5) $$g^{\prime }\left(g_{n}\right)=\alpha \arctan\left(\beta g_{n}\right)$$
- Tanh-type GPF:
  (6) $$g^{\prime }\left(g_{n}\right)=\alpha \tanh\left(\beta g_{n}\right)$$
- Log-type GPF:
  (7) $$g^{\prime }\left(g_{n}\right)=\alpha \left(\ln\left(\mathrm{ReLU}\left(\beta g_{n}\right)+1\right)-\ln\left(\mathrm{ReLU}\left(-\beta g_{n}\right)+1\right)\right)$$

There are two key parameters (α and β) in the GPF, which control the shape and strength of the gradient activation function, determining how GPF amplifies tiny gradients and limits large gradients. Specifically, α primarily controls the output range of the GPF, determining the maximum scaling factor applied to gradient values according to their magnitude. β mainly controls the sensitivity of GPF when gradient values are close to zero, thereby determining the steepness of the GPF curve in the region of small gradients. When β is large, the slope of GPF in the region near zero gradients becomes steeper, meaning that GPF significantly amplifies tiny gradients, facilitating faster parameter updates and mitigating the vanishing gradient problem.

## 4. Experiment

### 4.1. Datasets of Simulation

#### 4.1.1. Dataset 1

For the training dataset, we adopt the same training dataset as presented in [39], which is known as the CC dataset. The dataset is composed of both synthetic and real-world noisy images, which are detailed in the following. For the synthetic noise images, there are 400 images, presented as 180 × 180 pixel grayscale and color formats. To enhance model robustness, two data augmentation methods are employed. The first method scales the original images via bicubic interpolation with factors of 0.7, 0.8, 0.9, and 1. The second involves applying one of eight different geometric transformations, such as rotation and flipping. To ensure variability, each image undergoes only a single geometric transformation and is utilized a maximum of four times per epoch. For real noisy images, there are 100 JPEG compressed images with a resolution of 512 × 512 taken by five different cameras. Since these images are compressed, they will pose greater challenges to the image denoising algorithm.

To evaluate the model’s performance on real-world noise, experiments are conducted on the CC dataset [39]. The dataset is composed of 15 real-world noisy images of size 512 × 512, captured by three distinct digital cameras (i.e., Canon 5D Mark III, Nikon D600, and Nikon D800) under various high ISO settings (such as 1600, 3200, and 6400). Its use allows for a robust assessment of our model’s ability to handle complex and authentic noise patterns.

#### 4.1.2. Dataset 2

The dataset (known as the PolyU dataset) offers a standardized benchmark for real-world image denoising, comprising a curated collection of 100 high-resolution patches (512 × 512 pixels) sourced from 40 distinct natural scenes [40]. Captured across five different camera models, it provides a diverse representation of sensor-specific noise patterns under varying conditions. A key quantitative feature is its broad ISO range, which is more comprehensive than previous datasets, allowing for systematic evaluation of algorithm robustness [26]. The availability of meticulously aligned, high-quality reference (“ground truth”) images for each noisy sample enables precise performance metrics calculation, such as PSNR and SSIM, making it an essential resource for rigorous denoising research and validation.

### 4.2. Metrics

In order to quantitatively evaluate the denoising performance of AuroraNet on two datasets, we employ two standard metrics: Peak Signal-to-Noise Ratio (PSNR) [41] and Structural Similarity Index (SSIM) [42].

PSNR is a widely adopted objective metric in the field of image processing, which evaluates the fidelity of a reconstructed image by referencing its Mean Squared Error (MSE) relative to the original. A higher PSNR value indicates better image reconstruction quality and, thus, superior denoising performance. It is mathematically defined as

(8) $$\mathrm{PSNR}=10\cdot \log_{10}\left(\frac{{\left({\mathrm{MAX}}_{I}\right)}^{2}}{\mathrm{MSE}}\right),$$

where ${\mathrm{MAX}}_{I}$ denotes the maximum possible pixel value of the image (e.g., 255 for an 8-bit grayscale image). The MSE is defined as

(9) $$\mathrm{MSE}=\frac{1}{H\times W}\sum_{i=1}^{H}\sum_{j=1}^{W}{\left[I_{\mathrm{gt}}\left(i,j\right)-I_{\mathrm{denoised}}\left(i,j\right)\right]}^{2},$$

where *H* and *W* denote the height and width of the image, respectively. $I_{\mathrm{gt}}\left(i,j\right)$ represents the pixel value of the original, noise-free (ground truth) image at coordinates (i,j), while $I_{\mathrm{denoised}}\left(i,j\right)$ is the pixel value of the denoised image output by the model at the same coordinates.

SSIM evaluates the perceptual quality of the restored images. Unlike MSE, which calculates individual pixel-wise errors, SSIM focuses on the structural information of image windows, which aligns better with the human visual system. It measures the similarity between two images by combining three components: luminance, contrast, and structure. Given two corresponding windows *x* and *y* from the ground truth and the denoised image, the SSIM index is computed as follows:

(10) $$\mathrm{SSIM}\left(x,y\right)=\frac{\left(2\mu_{x}\mu_{y}+C_{1}\right)\left(2\sigma_{xy}+C_{2}\right)}{\left(\mu_{x}^{2}+\mu_{y}^{2}+C_{1}\right)\left(\sigma_{x}^{2}+\sigma_{y}^{2}+C_{2}\right)},$$

where $\mu_{x}$ and $\mu_{y}$ denote the mean intensity of windows *x* and *y*, respectively [42]. In addition, $\sigma_{x}^{2}$ and $\sigma_{y}^{2}$ are the variances of *x* and *y*, reflecting contrast, and $\sigma_{xy}$ is the covariance between *x* and *y*, indicating structural similarity. The constants $C_{1}={\left(k_{1}L\right)}^{2}$ and $C_{2}={\left(k_{2}L\right)}^{2}$ are introduced to maintain numerical stability when the denominator approaches zero. Here, *L* represents the dynamic range of pixel values (e.g., L=255 for 8-bit images), and the default coefficients are set to $k_{1}=0.01$ and $k_{2}=0.03$. The value of SSIM ranges from −1 to 1, where a value closer to 1 indicates higher similarity and better preservation of image details.

### 4.3. Validation of GPF on AuroraNet

To evaluate the performance of the GPF optimizer on complex real-world noise, a hybrid training dataset is constructed, including a large number of synthetic noisy patches generated from 400 clean images and real noisy patches extracted from 100 real noisy images. By training on such a diverse set of data, the model is expected to learn strategies for dealing with different types of noise simultaneously. In this simulation, the DudeNet model is trained using the GPF-enhanced SGD optimizer with three different activation functions (Arctan, Log, and Tanh), the standard Adam, and the SGD optimizer. Through multiple experiments, it has been found that the parameter sets {α=0.1,β=20} and {α=0.2,β=10} are relatively stable for the GPF-enhanced SGD optimizer. These values can serve as a starting point for initial attempts and fine-tuning.

The results are summarized in Table 1, where bold values in each row indicate the highest PSNR measurements, while underlined values denote the second-highest performance. The table comprehensively presents the PSNR values of each optimizer across diverse test images and their corresponding averages. In terms of mean performance, the GPF optimizer with Arctan activation (SGD_atanMom) demonstrated superior efficacy, achieving an average PSNR of 35.59 dB. This result not only exceeds the baseline SGD optimizer (35.02 dB) but also outperforms other GPF variants: SGD_log_Mom (35.32 dB) and SGD_tanh_Mom (35.17 dB). Notably, SGD_atanMom exhibits better performance than the widely adopted Adam optimizer (35.34 dB), which ranked second in this comparative analysis. Further examination of denoising outcomes across individual images reveals that SGD_atanMom consistently achieved either the highest or second-highest PSNR values in 82% of test cases, unequivocally demonstrating its competitive advantage and operational superiority. Therefore, Arctan activation is exploited in AuroraNet.

The introduction of the GPF in AuroraNet’s training demonstrates a significant improvement in both training stability and denoising performance. As shown in Table 1, the GPF optimizer with Arctan activation (SGD_atanMom) achieved an average PSNR of 35.59 dB. This result not only exceeds the baseline SGD optimizer (35.02 dB) but also outperforms the widely adopted Adam optimizer (35.34 dB). This highlights that the strategic modulation of gradient magnitudes by GPF is highly effective, leading to a more robust and efficient model.

The performance gains observed are attributed to the unique characteristics of the optimizer based on GPF. Unlike the standard Adam optimizer, which can be susceptible to issues like vanishing or exploding gradients during training, GPF dynamically adjusts the gradient to maintain a stable learning process. This mechanism allows the model to converge more efficiently and escape suboptimal local minima, thereby enabling AuroraNet to learn a more effective mapping for noise suppression while preserving critical image details. The improved PSNR and SSIM values are direct evidence of the ability of GPF to refine network parameters more precisely, which is crucial for achieving state-of-the-art denoising results.

**Figure 3:**
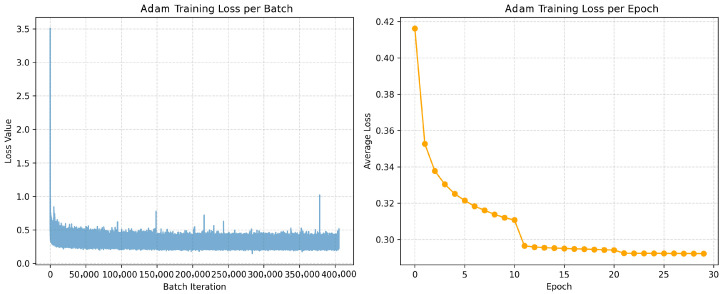
Adam optimizer loss function curve.

**Figure 4:**
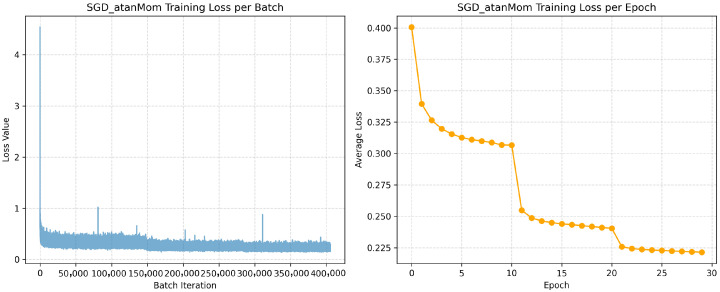
SGD_atanMom optimizer loss function curve.

To gain a deeper understanding of the specific impact of the GPF on the optimization process, the training loss dynamics of the standard Adam optimizer and SGD_atanMom are shown in Figure 3 and Figure 4, respectively, illustrating the training loss curves per batch and per epoch for the two optimizers. Comparing the left panels of Figure 3 and Figure 4, it is observed that the SGD_atanMom optimizer (GPF-SGD + Momentum) exhibits a relatively lower baseline fluctuation, indicating better instantaneous stability when processing each data batch. In contrast, the per-batch training loss for the Adam optimizer shows a slightly larger fluctuation range and some more prominent spikes. This phenomenon may suggest that, under the current hyperparameter settings, GPF’s mechanism of amplifying small gradients, combined with the inherent randomness of SGD, makes the per-batch loss more sensitive to the specific composition of the data batch. Optimizing the learning rate or other related hyperparameters for GPF-SGD + Momentum could be a potential way to further improve its per-batch loss stability.

For a deeper understanding of this phenomenon, it is useful to consult the existing literature on optimizer stability and the impact of stochastic gradients, such as the foundational work on Adam by Kingma and Ba [43] and on stochastic gradient descent by Bottou [38]. This discussion complements the conclusions in Table 1, demonstrating overall performance superiority of the GPF. Furthermore, the comparison in Figure 3 and Figure 4 reveals the specific dynamics of the training process as GPF achieves this advantage. It shows that GPF’s mechanism, while leading to excellent convergence, may also be accompanied by higher inter-batch loss fluctuations. This finding provides a direction for future research, which is how to enhance the training stability of GPF through fine-tuned hyperparameter optimization while retaining its convergence benefits.

Further observing the per-epoch average training loss curves (right panels of Figure 3 and Figure 4), both curves become very smooth, showing good macro training stability, without obvious signs of oscillation or divergence. Although both optimizers show rapid convergence in the initial stages, the loss value of the Adam optimizer tends to plateau after reaching a certain level, eventually stabilizing on a relatively high platform. On the other hand, the loss curve of SGD_atanMom demonstrates a continuous gradient descent trend and maintains a lower loss value throughout the training process, ultimately converging to a level below that of Adam (approximately 0.223 vs. 0.293). This continuous and effective gradient descent capability indicates that the GPF optimizer can better guide the model to overcome difficulties in the optimization process and more thoroughly minimize the loss function. The lower final training loss directly reflects a better fit of the model to the training data, which benefits from the more effective gradient descent optimization process of SGD_atanMom.

### 4.4. Comparison

**Figure 5:**
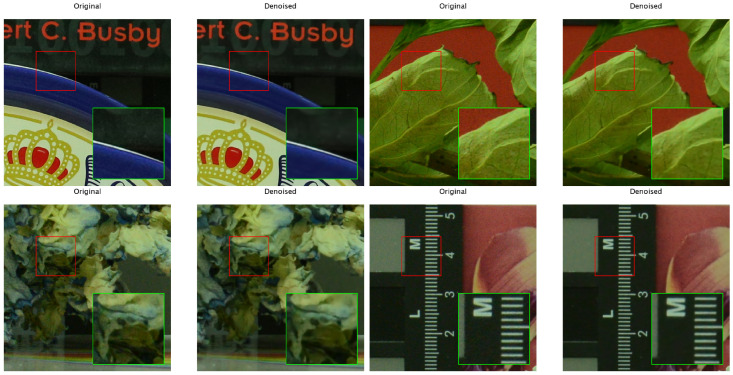
Image comparison before and after denoising.

To better demonstrate the superiority of AuroraNet, a comprehensive simulation that compares AuroraNet with various representative neural network models in the current image denoising field is performed. All methods were rigorously assessed on the CC real-noise dataset using PSNR as the primary metric. Results are shown in Table 2, where PSNR of various denoised images and their mean values are illustrated. According to Table 2, AuroraNet surpasses baseline models in processing unseen CC real-noise data. Specifically, AuroraNet achieved a mean PSNR of 35.59 dB on the CC dataset. Furthermore, qualitative comparisons (illustrating images pre- and post-denoising) are depicted in Figure 5, revealing that images processed by AuroraNet exhibit discernible advantages over baseline counterparts in terms of both fine-detail preservation and the suppression of visual artifacts. This visual evidence further corroborates the instrumental role of the GPF optimizer in augmenting the holistic performance and generalization proficiency of the DudeNet architecture when applied to practical real-world noise mitigation tasks.

These critical results are doubly significant in that they not only validate the effectiveness of the GPF under complex noise conditions but also confirm that GPF can enhance the generalization ability of the DudeNet model from training data to unknown real-world noise patterns. The inherent complexity of real-noise images—characterized by signal correlation, non-Gaussian distributions, and compression artifacts—typically induces rugged optimization landscapes in loss function spaces. GPF addresses this challenge through its non-linear gradient modulation capabilities, particularly its dual mechanism of amplifying critical small gradients while smoothing volatile large gradients. This adaptive adjustment enables the optimization process to more effectively learn noise-invariant priors related to image structure and content. By effectively modulating gradients, GPF allows AuroraNet to learn robust noise-invariant priors from simplistic synthetic data, which can then be successfully applied to complex real-world noise patterns. Consequently, AuroraNet achieves superior image reconstruction quality on dataset 1.

To further validate the applicability of the proposed model, comparative experiments were also conducted on dataset 2, whose results are shown in Table 3. The results in the table show that AuroraNet achieves clear gains over Dudenet on both PSNR and SSIM. Specifically, AuroraNet attains an average PSNR of 38.40 dB, slightly higher than the 38.06 dB obtained by Dudenet, indicating improved noise-suppression performance. Its advantage on SSIM is even more apparent: the score increases from 0.9601 to 0.9633, suggesting that AuroraNet offers better preservation of structural information and finer detail consistency. When compared with other widely used or high-performing deep denoising models, such as QNMF and NI [23,44], AuroraNet remains highly competitive, with overall performance reaching or approaching the top tier. Although R-REDNet achieves the highest PSNR and SSIM on this test set, AuroraNet delivers comparable image quality while using only about one-tenth of the parameters, underscoring the effectiveness and practical value of the proposed improvements.

## 5. Conclusions

This paper introduces AuroraNet, a novel deep learning model developed specifically for image denoising. Our approach innovatively combines the robust DudeNet architecture with a new optimizer based on GPF, addressing the limitations of traditional methods when dealing with complex, real-world noise. Through extensive experimental validation, we have reached the following key conclusions:

1. Effective Integration and Theoretical Alignment: We successfully built and deployed the AuroraNet model for image denoising. Its performance improvements and training enhancements align closely with the theoretical foundations of GPF. This provides a clear explanation for AuroraNet’s overall effectiveness.
2. Superior Denoising Performance: Our experimental results clearly show that AuroraNet significantly outperforms the baseline DudeNet and other state-of-the-art denoising algorithms. It achieves superior performance in key metrics such as PSNR and SSIM. This highlights the practical value of AuroraNet in enhancing image denoising quality.
3. Improved Training Dynamics and Generalization: A thorough analysis of the training loss curves and final test performance reveals that AuroraNet exhibits enhanced stability and convergence during training. Crucially, the gains observed on pure real noise test sets within a mixed training context strongly suggest that AuroraNet effectively learns from diverse data and possesses excellent generalization capabilities, allowing it to handle previously unseen noise conditions.
4. Strong Alignment Between Empirical Results and Theory: The performance improvements and training enhancements seen with AuroraNet closely align with the theoretical underpinnings of GPF. GPF is designed to mitigate common deep learning optimization issues, such as ill-conditioning, vanishing/exploding gradients, and difficulties escaping saddle points. This strong consistency provides a clear explanation for AuroraNet’s overall effectiveness.

In essence, this study demonstrates that AuroraNet provides an innovative solution for image denoising, capable of tackling complex real-world noise by strategically integrating the DudeNet architecture and the GPF optimizer. Driven by its novel optimization strategy, AuroraNet’s advanced capabilities in image denoising and fine detail preservation establish a new benchmark for deep learning-based image denoising. Future research could explore AuroraNet’s applicability to various image tasks and further refine its architecture to accommodate a broader spectrum of noise types.

## Figures and Tables

**Table 1:** PSNR (dB) of different optimizers. The best results are highlighted in bold, and the second-best results are indicated with underlining.

Camera Settings	Adam	SGD	SGD_atanMom	SGD_log_Mom	SGD_tanh_Mom
5dmark3_iso3200_1_real.png	36.31	35.79	**36.35**	36.27	35.54
5dmark3_iso3200_2_real.png	**36.82**	35.78	36.60	36.58	36.06
5dmark3_iso3200_3_real.png	34.95	34.57	34.54	**35.05**	34.42
d600_iso3200_1_real.png	**34.12**	33.77	33.55	33.81	33.65
d600_iso3200_2_real.png	**35.30**	34.53	34.52	33.84	33.70
d600_iso3200_3_real.png	37.69	37.15	**37.94**	37.03	37.28
d800_iso1600_1_real.png	37.49	37.37	**37.77**	37.63	37.68
d800_iso1600_2_real.png	37.63	37.47	38.00	37.94	**38.54**
d800_iso1600_3_real.png	35.75	34.52	**37.26**	35.74	35.77
d800_iso3200_1_real.png	36.26	35.92	**37.18**	36.59	35.93
d800_iso3200_2_real.png	34.56	34.97	**35.46**	35.13	35.16
d800_iso3200_3_real.png	36.93	37.08	**38.00**	37.37	37.25
d800_iso6400_1_real.png	31.93	32.07	32.01	**32.13**	32.07
d800_iso6400_2_real.png	32.07	31.83	**32.12**	32.10	32.01
d800_iso6400_3_real.png	32.35	32.45	32.59	**32.64**	32.49
average	35.34	35.02	**35.59**	35.32	35.17

**Table 2:** PSNR (dB) for different methods from public dataset 1. The best results are highlighted in bold, and the second-best results are indicated with underlining.

Camera Settings	AuroraNet	GAT-BM3D [15]	TID [16]	DnCNN [17]	DudeNet [9]
Canon 5D_iso3200_1	36.35	31.23	37.22	**37.26**	36.31
Canon 5D_iso3200_2	36.60	30.55	34.54	34.87	**36.82**
Canon 5D_iso3200_3	34.54	27.74	34.25	34.09	**34.95**
d600_iso3200_1	33.55	28.55	32.99	33.62	**34.12**
d600_iso3200_2	34.52	32.01	34.20	34.48	**35.30**
d600_iso3200_3	37.94	**39.78**	35.58	35.41	37.69
d800_iso1600_1	37.77	32.24	34.49	**37.95**	37.49
d800_iso1600_2	**38.00**	33.86	35.19	36.08	37.63
d800_iso1600_3	**37.26**	33.90	35.26	35.48	35.75
d800_iso3200_1	**37.18**	36.49	33.70	34.08	36.26
d800_iso3200_2	**35.46**	32.91	31.04	33.70	34.56
d800_iso3200_3	38.00	**40.20**	33.07	33.31	36.93
d800_iso6400_1	**32.01**	29.84	29.40	29.83	31.93
d800_iso6400_2	**32.12**	27.94	29.86	30.55	32.07
d800_iso6400_3	**32.59**	29.15	29.21	30.09	32.35
average	**35.59**	32.43	33.33	34.05	35.34

**Table 3:** Average results on PSNR (dB) and SSIM among different models on the 100 cropped images in dataset 2. The best results are highlighted in bold, and the second-best results are indicated with underlining.

Metric	Models						
	Dudenet [9]	AuroraNet	DnCNN [17]	QNMF [44]	QWSNM [45]	NI [23]	R-REDNet [26]
PSNR	38.06	38.40	36.08	38.10	37.88	37.77	**44.01**
SSIM	0.9601	0.9633	0.9161	0.9625	0.9612	0.9570	**0.9931**

## Data Availability

The original contributions presented in this study are included in the article. Further inquiries can be directed to the corresponding author.
